# Concordance between Response Assessment Using Prostate-Specific Membrane Antigen PET and Serum Prostate-Specific Antigen Levels after Systemic Treatment in Patients with Metastatic Castration Resistant Prostate Cancer: A Systematic Review and Meta-Analysis

**DOI:** 10.3390/diagnostics11040663

**Published:** 2021-04-07

**Authors:** Sangwon Han, Sungmin Woo, Yong-il Kim, Jae-Lyun Lee, Andreas G. Wibmer, Heiko Schoder, Jin-Sook Ryu, Hebert Alberto Vargas

**Affiliations:** 1 Department of Nuclear Medicine, Asan Medical Center, University of Ulsan College of Medicine, Seoul 05505, Korea; hswon87@naver.com (S.H.); kyi821209@naver.com (Y.-i.K.); jsryu2@amc.seoul.kr (J.-S.R.); 2 Department of Radiology, Memorial Sloan Kettering Cancer Center, New York, NY 10065, USA; wibmera@mskcc.org (A.G.W.); schoderh@MSKCC.ORG (H.S.); vargasah@mskcc.org (H.A.V.); 3 Department of Oncology, Asan Medical Center, University of Ulsan College of Medicine, Seoul 05505, Korea; jaelyun@amc.seoul.kr

**Keywords:** positron emission tomography, prostate-specific antigen, prostate specific membrane antigen, response assessment, metastatic castration-resistant prostate cancer

## Abstract

Prostate-specific membrane antigen positron emission tomography (PSMA PET) has recently gained interest as a promising tool for treatment response evaluation in metastatic castration-resistant prostate cancer (CRPC). We performed a systematic review and meta-analysis assessing the concordance between response evaluation using PSMA PET and serum prostate-specific antigen (PSA) level after systemic treatment and the association between PSMA PET and overall survival in metastatic CRPC patients. PubMed, Embase, and Cochrane library databases were searched until August 2020. Studies that reported the concordance between PSMA PET and PSA response were included. PSMA PET and PSA response evaluation were dichotomized into response vs. non-response to construct two-by-two contingency tables; an ≥30% increase in PSMA PET according to PET Response Criteria in Solid Tumors 1.0 and as an increase in serum PSA level of ≥25% as per Prostate Cancer Working Group 3 guidelines were defined as non-response. The percent agreement rates were pooled using random-effect model. Ten studies (268 patients) were included. The concordance rates ranged 0.50–0.84 with a pooled proportion of 0.73 (95% confidence interval 0.67–0.79). Patients were treated with ^177^Lu-PSMA therapy in five, chemotherapy in three, ^223^Ra in one, and more than one type in one study. Various PET parameters were used: the most widely evaluated was PSMA tumor volume (PSMA-TV). Similar proportions were found across different therapeutic agents, PET response parameters, and regarding directionality of discordance (PSA response/PSMA non-response vs. PSMA response/PSA non-response). Two studies reported that a decrease in PSMA-TV was associated with better overall survival. PSMA PET and PSA response assessments were discordant in nearly a fourth of metastatic CRPC patients. Further studies are warranted to establish the clinical meaning of this discordance and define appropriate management for such clinical situation.

## 1. Introduction

Prostate cancer is the most frequently diagnosed cancer in men and the second leading cause of cancer-specific deaths [1]. Although it is initially sensitive to androgen deprivation therapy, it subsequently develops into a castration-resistant state. Castration-resistant prostate cancer (CRPC), especially when metastatic, is considered incurable and accounts for most prostate cancer-related deaths. Nevertheless, several approved and investigational systemic drugs such as enzalutamide, abiraterone, and ^223^Ra have shown a survival benefit in clinical trials [2,3,4], and it has become even more important to monitor response to these therapies for deciding on further management.

Currently, response assessment for systemic treatments in patients with metastatic CRPC is primarily done using conventional imaging and biochemical testing—that is, Response Evaluation Criteria in Solid Tumors (RECIST) on CT, bone scan, and serum prostate-specific antigen (PSA) [5,6]. However, serum PSA levels have demonstrated limitations with regards to their ability to accurately assess therapeutic response and do not necessarily correlate with survival. In addition, there is difficulty in interpreting the significance of changes in bone metastases using CT and bone scan, the latter reflecting osteoblastic response in bone rather than the metastatic tumor itself. Therefore, newer methods to determine therapeutic response have been investigated including molecular imaging [7,8] and circulating tumor cells [9].

Prostate specific membrane antigen (PSMA) positron emission tomography/computed tomography (PET/CT) has the capability to detect metastatic disease more accurately than conventional imaging in both the recurrent and primary setting [10,11], substantially impacting the management of patients with prostate cancer [12]. A baseline PSMA PET is also highly recommended in patients with CRPC for accurate assessing of disease extent (i.e., non-metastatic, oligometastatic vs. polymetastatic) [13] and appropriate selection of therapeutic strategy including metastasis-derived therapy or systemic treatment [14]. Based on this, the notion of PSMA PET being used for response assessment has gained interest in recent years as shown in multiple recent papers that have used PSMA PET/CT or PET/magnetic resonance imaging (MRI) for this purpose [15,16,17,18,19,20,21,22,23,24], in proposals for standardized PSMA PET progression criteria [7], and in the consensus statement by the European Association of Urology (EAU) and European Association of Nuclear Medicine (EANM) in 2020 [8]. Currently available literature on PSMA PET response mainly evaluates its concordance with PSA response, whereas only a few studies investigated its direct association with survival [15,16,17,18,19,20,21,22,23,24]. To address this, we performed a systematic review and meta-analysis to assess the concordance between response evaluation using PSMA PET and PSA after systemic treatment and the association between PSMA PET and other robust endpoints of overall and radiologic progression-free survival in patients with metastatic CRPC.

## 2. Materials and Methods

### 2.1. Literature Search

This meta-analysis was performed according to the PRISMA guidelines and the protocol was registered in PROSPERO (CRD42020206349) [25]. PubMed, Embase, and Cochrane library databases were searched until 27 August 2020. The search query was formed using the following keywords: prostate cancer, PSMA PET, and response (Supplementary Table S1). All similar articles suggested on above websites and the reference lists of the identified papers were scrutinized to additionally find eligible studies.

### 2.2. Inclusion Criteria

Studies were included if they satisfied the following patient/index test/comparator/outcome/study design (PICOS) criteria: (1) patients (P) with metastatic CRPC; (2) PSMA PET, PET/CT or PET/MRI used as the index test (I); (3) PSA used as the comparator (C); (4) concordance between response assessment between PSMA PET and PSA as the outcome (O); and (5) randomized trials or any type of prospective or retrospective cohort studies as the study design (S).

### 2.3. Exclusion Criteria

Studies were excluded if they met one of the following criteria: (1) included fewer than 10 patients; (2) patient populations differed from our research questions (e.g., castration sensitive prostate cancer, non-metastatic disease, or non-systemic therapeutic modalities such as stereotactic radiation treatment); and (3) focused on the “flare” phenomenon of increased PSMA uptake to investigate the short-term effects of androgen deprivation therapy [26]. No language restrictions were applied. If any studies had overlapping study populations, we planned to include the study with the higher level of evidence (e.g., prospective over retrospective; multicenter over single center; and larger over smaller study population) and more detailed information provided (e.g., individual patient data, used more types of response assessment criteria). Two reviewers (S.H. and S.W.) independently performed the literature search and study selection. Consensus was reached after discussion with a third reviewer (H.A.V.).

### 2.4. Data Extraction

The following information regarding study and PET characteristics were extracted from each study in a standardized manner: (1) study characteristics—publication information (journal and year), origin of study (institution and country), study design, number and median/mean age of patients, type of systemic treatment, and pretreatment median/mean PSA level and distribution of Gleason scores; (2) PET characteristics—type of radiotracer, injected activity, uptake time, equipment, timing of PET acquisition, and criteria used for response assessment on PET and by PSA level, respectively.

### 2.5. Quality Assessment

The quality of the studies was rated using the Quality Assessment of Diagnostic Accuracy Studies-2 (QUADAS-2) tool [27] which assesses the risk of bias and applicability concerns in the following four domains: patient selection, index test, reference standard, and flow and timing. For the purpose of quality assessment, the comparator PSA was regarded as the reference standard. The same two reviewers (S.H. and S.W.) independently performed both data extraction and quality assessment followed by discussion with a third reviewer (H.A.V.) in case of disagreement.

### 2.6. Data Synthesis and Analysis

Data for PSMA PET response evaluation were categorized as complete response (CR), partial response (PR), stable disease (SD), and progressive disease (PD) according to PET Response Criteria in Solid Tumors (PERCIST) 1.0 in all the included studies but one using European Organization for Research and Treatment of Cancer (EORTC) classification which set ±30% (PERCIST 1.0) and ±25% (EORTC) changes in size or uptake as cut-offs for discriminating PR/SD/PD, respectively, and then were dichotomized to response (CR, PR, and SD) vs. non-response (PD) to build 2 × 2 contingency tables [7,8]. Therefore, non-response (PD) was defined as appearance of new lesions or increase ≥30% (PERCIST 1.0) or ≥25% (EORTC) in size or uptake in the absence of new lesions on PSMA PET. Regarding PSA response, we dichotomized the data in the same manner (CR, PR, and SD vs. PD) using the definition of PD as an increase in serum PSA level of ≥25% from start of therapy to time of second PET/CT as per Prostate Cancer Working Group 3 (PCWG3) guidelines [6]. Afterwards, we constructed 2 × 2 contingency tables comparing PSMA PET and PSA as follows: (1) PSA response/PSMA response; (2) PSA response/PSMA non-response; (3) PSA non-response/PSMA response; and (4) PSA non-response/PSMA non-response. When more than one type of response-assessment criteria was used for PSMA PET evaluation, we planned to select the one recommended by consensus statements and most used among the included studies, for the purpose of overall meta-analytical pooling [7].

The primary outcome was the concordance in terms of percent agreement rate between PSA and PSMA response. Secondary outcomes were (1) subgroup analyses of the concordance rates stratified to PET response assessment criteria and therapeutic agent, (2) directionality of the discordant responses between PSA and PSMA, and (3) correlation between PSMA response assessment and clinical outcomes such as overall and radiologic progression-free survival. Concordant and discordant proportions were transformed using the Freeman-Tukey double arcsine method [28] and were then meta-analytically pooled using the DerSimonian-Liard method for calculating weights with the meta and metafor packages in R software (version 3.6.3; R Foundation for Statistical Computing, Vienna, Austria) with *p* < 0.05 indicating statistical significance. Clopper-Pearson confidence intervals (CIs) were used for individual studies. Higgins *I*^2^ statistics were used to assess heterogeneity [29]. Funnel plots with Egger’s test were drawn to appraise the presence of publication bias [30].

## 3. Results

### 3.1. Literature Search

**Figure 1:**
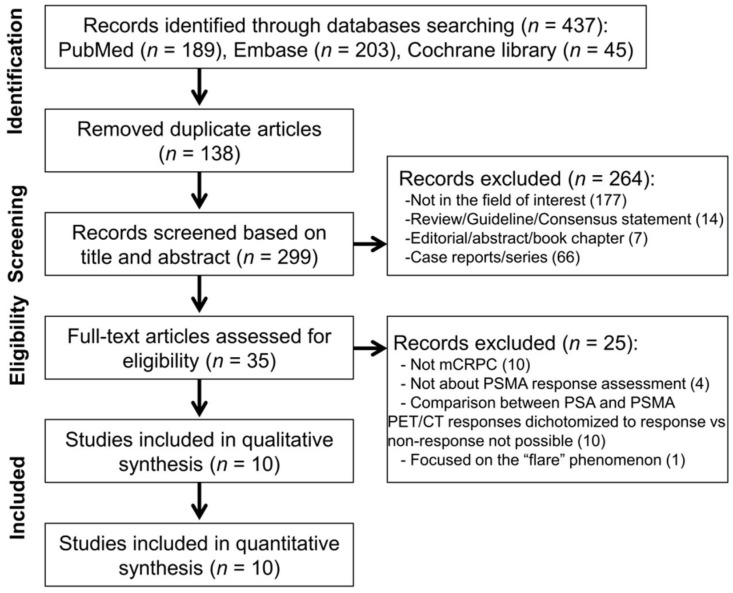
PRISMA flow chart showing the study selection process. CRPC = castration-resistant prostate cancer.

The detailed PRISMA study selection process is shown in Figure 1. The initial systematic search found 437 studies, and after the removal of 138 duplicate articles, a total of 299 articles were screened based on their titles and abstracts. Of these article, 35 were selected for full-text, among which 25 were excluded due to the following reasons: (1) not metastatic CRPC (*n* = 10); (2) did not assess response on PSMA PET (*n* = 4); (3) comparison between PSA and PSMA PET responses dichotomized to response vs. non-response not possible (*n* = 10); and (4) focused on the flare phenomenon (*n* = 1). Ultimately, ten studies were included in our meta-analysis [15,16,17,18,19,20,21,22,23,24].

### 3.2. Characteristics of Included Studies

Study and PET characteristics are described in Table 1 and Table 2. In brief, study design was retrospective in all ten studies. The radiotracers were ^68^Ga-PSMA-11 or ^68^Ga-PSMA-I&T. The therapeutic agents included ^177^Lu-PSMA radionuclide therapy in five, taxane-based chemotherapy in three, ^223^Ra radionuclide therapy in one, and mixed in one. PSMA PET was performed after about three cycles or completion of radionuclide therapy or chemotherapy. Regarding new-generation antiandrogen therapy, PSMA PET was performed at three months after the start of treatment. Various PET response criteria were used in the included studies: among them, the most widely evaluated was PSMA tumor volume (PSMA-TV, selected as representative PSMA PET response evaluation criterion for meta-analytical purposes in studies that provided results for multiple criteria), followed by SUVmax, summed SUVmean, and total lesion PSMA (TL-PSMA, which is a product of PSMA-TV and SUVmean of each lesion) with a threshold of PR, SD, PD as −30, and +30% changes suggested by PERCIST 1.0 classification.

### 3.3. Quality Assessment

**Figure 2:**
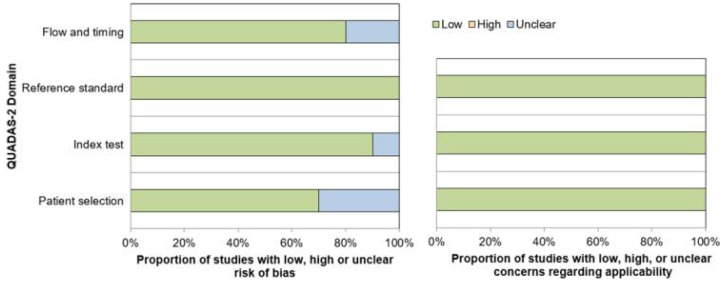
Quality Assessment of Diagnostic Accuracy Studies-2 (QUADAS-2) quality assessment of the 10 included studies.

The overall quality of the studies was considered good (Figure 2). Regarding the patient selection domain, three studies had unclear risk of bias as these studies were retrospectively designed and it was not clear whether patients were consecutively enrolled [20,23,24]. Regarding the index test domain, there was unclear risk of bias in one study because the definition of PET response criterion was unclear [16]. Regarding the flow and timing domain, two studies had unclear risk of bias as the interval between PSA and PSMA PET response evaluation was not reported [19,21]. All studies were of low risk of bias in the reference standard domain and had low concerns for applicability in all domains.

### 3.4. Concordance Rate between PSA and PSMA Response

**Figure 3:**
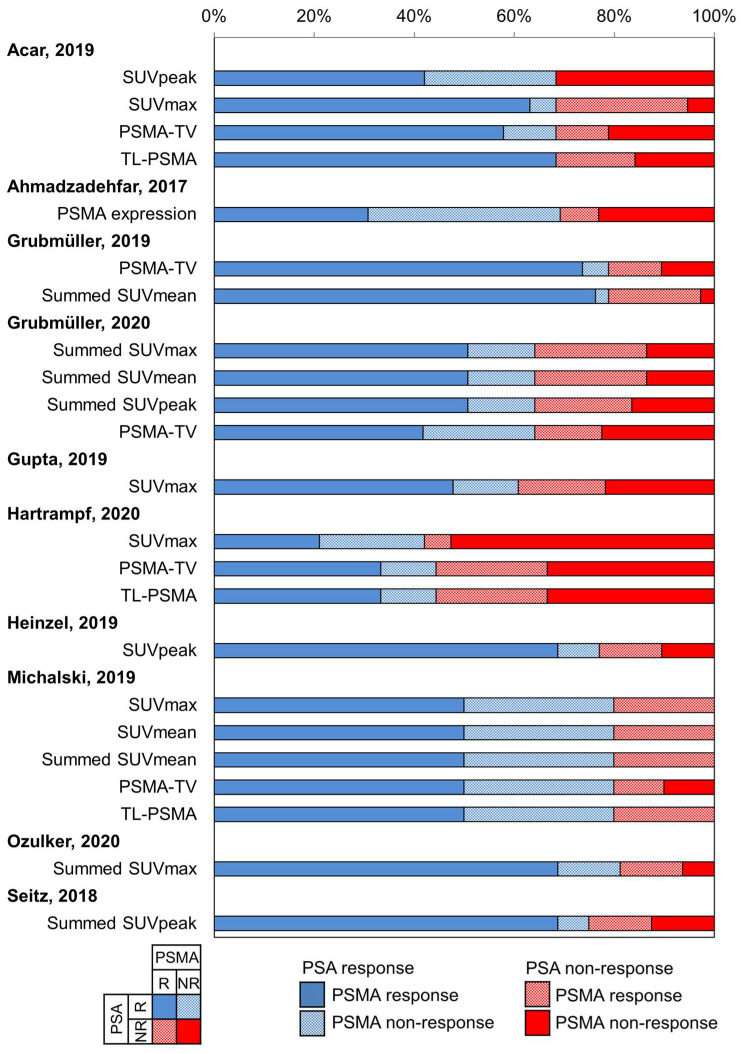
Breakdown of prostate-specific antigen (PSA) and prostate-specific membrane antigen positron emission tomography (PSMA PET) response assessment. NR = non-response; PSMA-TV = PSMA tumor volume; R = response; SUV = standardized uptake value; TL-PSMA = total lesion PSMA.

**Figure 4:**
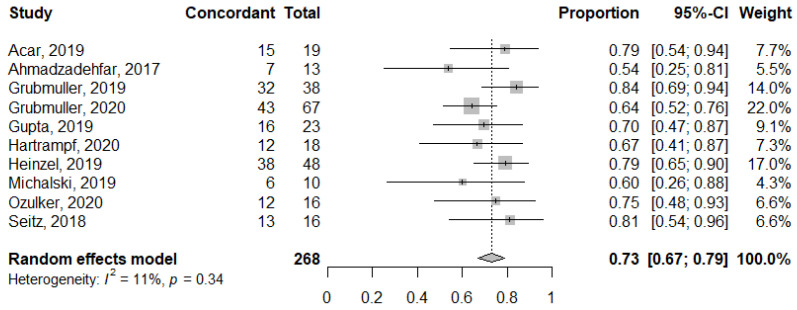
Forest plot of the concordant proportion (concordant/total number of patients) between PSA and PSMA PET response assessments.

**Figure 5:**
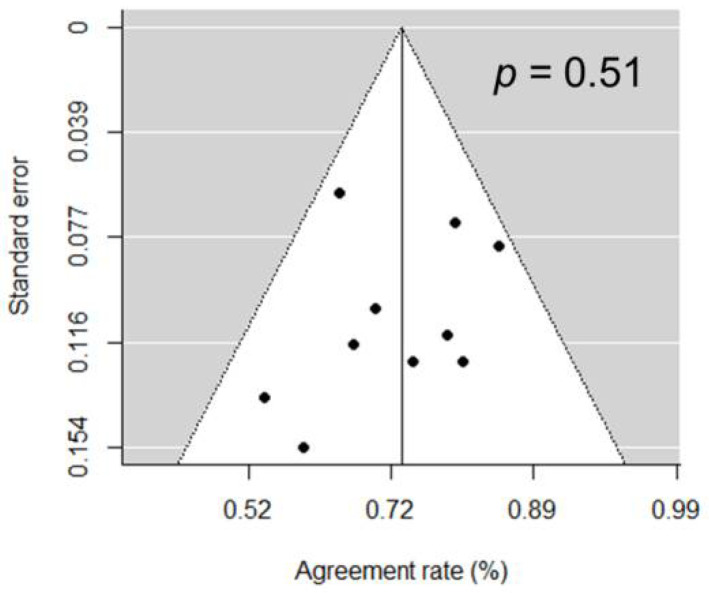
Funnel plot of the 10 included studies suggesting no publication bias being present.

The concordance rates between PSA and PSMA response ranged from 0.50 to 0.84; the 2 × 2 breakdown of the response categories (response vs. non-response) for both PSA and PSMA are summarized in Figure 3. The pooled proportion for all 10 studies was 0.73 (95% CI 0.67–0.79; Figure 4). There was no substantial heterogeneity between the studies (*I*^2^ = 0%). Publication bias was not present based on the funnel plot and Egger’s test (*p* = 0.51; Figure 5).

Subgroup analysis according to the type of therapy and PSMA PET criteria are summarized in Table 3. There were no significant differences in the pooled proportion of concordant response when stratified to type of therapeutic agent (*p* = 0.12): 0.78 (95% CI 0.71–0.85) in five studies using ^177^Lu radionuclide therapy; 0.74 (95% CI 0.61–0.86) in three studies using chemotherapy; 0.54 in one study that used ^223^Ra; and 0.64 in one study that used more than one type of therapeutic agent. There was no significant difference in the pooled proportions when stratified to PET response assessment criteria (*p* = 0.65): 0.72 (95% CI 0.62–0.82) in five studies using PSMA-TV; 0.68 (95% CI 0.56–0.79) in four using SUVmax; 0.68 (0.54–0.81) in three using summed SUVmean; and 0.70 (95% CI 0.50–0.87) in three using TL-PSMA.

### 3.5. Directionality of Discordant Responses between PSA and PSMA

**Figure 6:**
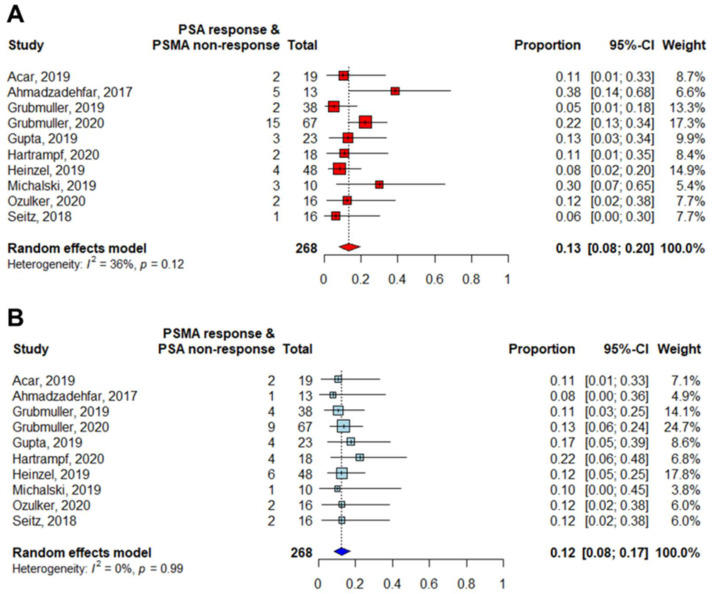
Forest plots of the proportions (discordant/total number of patients) of discordant responses between PSA and PSMA PET: (**A**) PSA response and PSMA non-response. (**B**) PSMA response and PSA non-response.

PSA and PSMA were similarly discordant in both directions (*p* = 0.82): The pooled proportion of patients that were responders on PSA but non-responders on PSMA PET was 0.13 (95% CI 0.08–0.20), while that of patients who were non-responders on PSA but responders on PSMA was 0.12 (95% CI 0.08–0.17), as shown in Figure 6.

### 3.6. Correlation between PSMA Response and Clinical Outcomes

Four of the included studies reported correlation between PSMA response and overall survival while there were no studies assessing the correlation between PSMA response and radiologic progression-free survival. Acar et al. [15] reported that a decrease in PSMA-TV or TL-PSMA was associated with better overall survival, while a decrease in SUVpeak, SUVmax, or serum PSA level was not in patients undergoing ^177^Lu radionuclide therapy. Grubmüller et al. [17] reported significantly longer overall survival in responders based on both PSMA-TV or serum PSA level, whereas summed SUVmean and RECIST evaluation were not associated with overall survival in patients using ^177^Lu radionuclide therapy. In contrast, in another study by Grubmüller et al. [18], where patients were treated with mixed therapeutic agents, neither PET (using PSMA-TV, summed SUVmax, summed SUVmean, summed SUVpeak), RECIST, nor PSA evaluation were associated with overall survival. Finally, Heinzel et al. [21] found that a decline in SUVpeak > 30% to ^177^Lu radionuclide therapy was not associated with overall survival.

## 4. Discussion

In this meta-analysis, we investigated the agreement between PSMA PET and PSA for response assessment in ten studies that evaluated patients with metastatic CRPC treated systemically. Almost a fourth of patients (27%) showed discordance between the two tests. The inherent limitations of PSA and conventional imaging including CT and bone scan for determining treatment response are well known and together with the capability of PSMA PET to better identify presence, sites, and burden of metastatic tumor in CRPC, this discrepancy opens up opportunities for potentially better response assessment that may result in better therapeutic planning (continuing, changing, or adding type of therapies) with the hopes of achieving better survival. With the currently available data, it is not possible to establish whether PSMA PET is superior to PSA as a response marker, and we cannot completely grasp the clinical significance of the discordance between the two. For example, it remains unanswered what course of action should be taken when there is PSA response but lack of response on PSMA PET, or vice versa. However, few of the included studies additionally showed that response on PSMA PET was associated with overall survival while PSA was not, indicating the potential superiority of PSMA PET-based response assessment [15,17]. In the context of clinical trials, PSMA PET should be encouraged for the purpose of response assessment in metastatic CRPC (especially as discordance cannot be predicted a priori at the moment) enabling future studies to answer and validate the clinical meaning of this discordance and their correlation with patient outcomes.

A multitude of different parameters and response assessment criteria were used across the ten included studies, probably because no clear response parameter for PSMA PET have been defined. In the limited sample we investigated, there were no significant differences between the various parameters and criteria with regards to their concordance with PSA response, and both PSMA uptake and volumetric measures may be useful for determining response in patients with metastatic CRPC. Nevertheless, it is conceivable that volumetric PET measurements in this patient population could provide additional value due to several reasons: (1) volumetric parameters like PSMA-TV or TL-PSMA capture the overall tumor burden [31]; (2) the degree of PSMA uptake and extent of metastases have both been shown to be correlated with survival in metastatic CRPC [32]; (3) volumetric measurements can be reproducibly performed using commercially available software [20]; and (4) tumor volumetry on PET have achieved encouraging results in different cancers [33,34]. Nevertheless, the biologic meaning of each PSMA response parameter is not fully understood. For example, a preclinical study found that PSMA expression of tumor cells did not change after taxane-based chemotherapy, and that tracer uptake was proportional to the number of viable cells [35]. Nevertheless, further studies (both in vitro, in vivo, and clinical) are needed to establish the differences in the meaning of each parameter and how they can be differentially incorporated in response assessment for different types of treatments.

The discordance in response assessment based on PSMA PET and PSA were similarly distributed in both directions. Specifically, the proportion of patients showing response on PSMA PET but non-response with PSA was 12% (95% CI 8–17%) while the opposite (non-response on PSMA PET and response with PSA) was 13% (95% CI 8–20%). Although not directly deducible from the included studies, we proposed the following possible explanations for these discordances: (1) There could be inherent phenotypic differences between different clones emerging during progression in terms of their expression of PSMA- and PSA-related genes [36]. (2) Mixed responses may occur between different metastatic sites, for example, a decrease in size of most tumors but increase in size (or even new appearance) in the minority of tumors [37]. This could result in an overall decrease in the PSA, but even one new metastatic foci would be considered progressive disease on PSMA PET based on assessment criteria by all but one of the included studies [16,17,18,19,20,21,22,23,24]. (3) Neuroendocrine or other forms of dedifferentiation of prostate cancer can manifest with low or variable levels of expression of PSA and PSMA [38]. (4) Additionally, pseudoprogression and flare phenomena could affect the relationship between PSMA and PSA response assessments [37,39,40].

Several limitations of our study deserve consideration. First, the sample size was relatively small for a meta-analysis, and all included studies were retrospective in design. This is probably because response assessment using PSMA PET is still in its early steps and has not been well established compared with other harder endpoints such as overall and radiologic progression-free survival. Nevertheless, this constitutes the largest body of evidence with the highest level of evidence until now. Second, although we were able to assess the overall concordance (or discordance) between PSMA PET and PSA for response assessment, we were unable to answer the clinical implications for this discordance, such as, when discordance is expected, what steps to take when we encounter such discordance, and correlation with patient outcomes. This was beyond the scope of our meta-analysis and will need to be investigated in future studies. Third, although we did not find significant difference in the concordance between PSMA PET and PSA between subgroups stratified to type of treatment, we cannot come to a hard conclusion for some treatments that only were used in a small number of studies (e.g., ^223^Ra radionuclide therapy in only one study).

## 5. Conclusions

PSA and PSMA PET response assessments are discordant in nearly a fourth of patients with metastatic CRPC undergoing systemic treatments. Results were consistent across different therapeutic agents and PET response criteria. Further studies are needed to elucidate the importance of this discordance and how to manage patients when encountering such clinical situations.

## Figures and Tables

**Table 1:** Study characteristics.

Author	Journal	Year	Institution	Country	Period	Design	Treatment	*n*	Pretreatment PSA (Median, ng/mL)	Initial Gleason Score (Median, Range)
Acar [15]	Ann Nucl Med	2019	Ataturk Training and Research Hospital	Turkey	2015–2018	R	^177^Lu-PSMA I&T	19	NR	9 (6–10)
Ahmadzadehfar [16]	J Nucl Med	2017	University Hospital Bonn	Germany	2014–2016	R	^223^Ra	13	NR	8 (6–10)
Grubmüller [17]	Eur J Nucl Med Mol Imaging	2019	Medical University of Vienna	Austria	2015–2018	R	^177^Lu-PSMA 617	38	60.8 (IQR 15.4–264.2)	NR
Grubmüller [18]	The prostate	2020	Medical University of Vienna	Austria	2015–2019	R	^223^Ra/cabazitaxel/docetaxel abiraterone/enzalutamide	43 *	11.3 (IQR 3.3–30.1)	NR
Gupta [19]	Urol Ann	2019	Rajiv Gandhi Cancer Insttute and Research Centre	India	NR	R	^177^Lu-PSMA 617	23	66 (5.4–550)	7 (6–9)
Hartrampf [20]	J Clin Med	2020	University Hospital Würzburg	Germany	2014–2018	R	docetaxel/cabazitaxel	21	15.0 (0–800)	8 (6–10)
Heinzel [21]	Eur J Nucl Med Mol Imaging	2019	university hospitals of Bonn and Aachen	Germany	2014–2018	R	^177^Lu-PSMA 617	48	NR	NR
Michalski [22]	Nuklearmedizin	2019	University of Freiburg	Germany	2015–2018	R	^177^Lu PSMA-617	10	812.1 ± 972.4	8 (7–9)
Ozulker [23]	Rev Esp Med Nucl Imagen Mol	2020	Okmeydanı Training and Research Hospital	Turkey	2017–2018	R	docetaxel	16	61.37 ± 102.2	8.50 ± 0.97
Seitz [24]	Eur J Nucl Med Mol Imaging	2018	Technical University of Munich	Germany	2013–2016	R	docetaxel	16	28 (2–3176)	≤6: 1 7: 6 ≥8: 7

* 43 patients undergoing 67 systemic therapies; IQR = interquartile range; NR = not reported; PSA = prostate-specific antigen; R = retrospective.

**Table 2:** Study characteristics.

Author	Tracer	Injected Dose (MBq)	Uptake Time (min)	Machine	PET Timing	PET Response Criteria
Acar [15]	^68^Ga-PSMA-I&T	115 *	60	PET/CT	after last cycle	SUVpeak SUVmax PSMA-TV TL-PSMA
Ahmadzadehfar [16]	^68^Ga-PSMA-11	2/kg	77 ^†^	PET/CT	after last cycle	PSMA expression (not explicit)
Grubmüller [17]	^68^Ga-PSMA-11	2/kg	60	PET/CT or PET/MRI	after 3 cycle	PSMA-TV Summed SUVmean
Grubmüller [18]	^68^Ga-PSMA-11	2/kg	60	PET/CT or MRI	^223^Ra or systemic Tx.: after 6 cycle abiraterone or enzalutamide: at 3 mo.	Summed SUVmax Summed SUVmean Summed SUVpeak PSMA-TV
Gupta [19]	^68^Ga-PSMA-11	2/kg	60	PET/CT	after radionuclide therapy	SUVmax
Hartrampf [20]	^68^Ga-PSMA-I&T	119–125 *	71–82 ^†^	PET/CT	after completion or termination of chemotherapy	SUVmax PSMA-TV TL-PSMA
Heinzel [21]	^68^Ga-PSMA-11	2/kg	60	PET/CT	after 3 or 4 cycle	SUVpeak
Michalski [22]	^68^Ga-PSMA-11	206 *	60	PET/CT	after 2 cycle	SUVmax SUVmean Summed SUVmean PSMA-TV TL-PSMA
Ozulker [23]	^68^Ga-PSMA-I&T	2/kg	60	PET/CT	after ≥3 cycles	Summed SUVmax ^‡^
Seitz [24]	^68^Ga-PSMA-11	154 *	57 *	PET/CT	after 3 cycle	Summed SUVpeak

* mean; ^†^ Applicable, as individual patient data were available; ^‡^ An increase in 25% in accordance with EORTC criteria was defined as non-response. All the other studies set +30% following PERCIST 1.0; PSMA-TV = PSMA tumor volume; RECIST = The Response Evaluation Criteria in Solid Tumors; SUV = standardized uptake value; TL-PSMA = total lesion PSMA.

**Table 3:** Subgroup analysis of concordant proportion between PSA and PSMA PET response assessments stratified to therapeutic agent and PSMA PET criteria.

Variables	Subgroups	Studies (*n*)	Pooled Proportion	95% CI	*I* ^2^	*p* *
Therapeutic agents	^177^Lu-PSMA	5	0.78	0.71–0.85	0%	0.69
	Chemotherapy	3	0.74	0.61–0.86	0%	
PET criteria	PSMA-TV	5	0.72	0.62–0.82	36%	0.33
	SUVmax	4	0.68	0.56–0.79	0%	
	Summed SUVmean	3	0.68	0.54–0.81	50%	
	TL-PSMA	3	0.70	0.50–0.87	45%	

Subgroups containing ≥3 studies were shown; * *p*-values of test for subgroup differences; CI = confidence interval; NA = not applicable; PSMA-TV = PSMA tumor volume; SUV = standardized uptake value; TL-PSMA = total lesion PSMA.

## Data Availability

Data is contained within the article or supplementary material.

## Supplementary Materials

The following are available online at https://www.mdpi.com/article/10.3390/diagnostics11040663/s1, Table S1: The queries and results of electronic search on PubMed, Embase, and Cochrane Library.
