# Supplementary material for: Concordance between Response Assessment Using Prostate-Specific Membrane Antigen PET and Serum Prostate-Specific Antigen Levels after Systemic Treatment in Patients with Metastatic Castration Resistant Prostate Cancer: A Systematic Review and Meta-Analysis
Source: Diagnostics (Basel). 2021 Apr 7;11(4):663. doi: 10.3390/diagnostics11040663 (PMC8067707; doi:10.3390/diagnostics11040663)
Supplement: Supplementary file 1 [file diagnostics-11-00663-s001.pdf]

Table S1. The queries and results of electronic search on PubMed, Embase, and Cochrane Library.

PubMed; Date: 27 August 2020

PICOS	#	Search Queries	Articles #
P-KQ1	**1**	((Prostatic Neoplasms[MeSH Terms]) OR (prostat* AND (tumor OR tumour OR cancer OR carcinoma OR malignan* OR neoplasm*))) [All Fields]	186,341
I	**2**	((Positron-Emission Tomography[MeSH Terms]) OR (“positron-emission tomography” OR “positron emission tomography” OR PET OR PET?CT OR PET/CT OR PET-CT OR PET?MRI OR PET?MR OR PET/MRI OR PET/MR OR PET-MRI OR PET-MR)) [All Fields]	135,539
	**3**	(PSMA OR "prostate specific membrane antigen") [All Fields]	2,428
I-KQ1	**4**	#2 AND #3 Sangwon Han:	1,790
P&I KQ1	**5**	#1 AND #4	1,675
O	**6**	response [All Fields]	2,813,142
KQ1 limit	**7**	#5 AND #6	217
case report	**8**	Case Reports [Publication Type]	
Final KQ1	**9**	#7 NOT #8	189

Embase; Date: 27 August 2020

PICOS	#	Search Queries	Articles #
P-KQ1	**1**	"prostate cancer"/mj OR (prostat* AND (tumor OR tumour OR cancer OR carcinoma OR malignan* OR neoplasm*)):ti,ab,kw	245,505
I	**2**	"positron emission tomography"/exp OR (“positron-emission tomography” OR “positron emission tomography” OR PET OR “PET CT” OR PET*CT OR PET-CT OR “PET MRI” OR “PET MR” OR PET*MRI OR PET*MR OR PET-MRI OR PET-MR):ti,ab,kw	249,576
	**3**	(PSMA OR "prostate specific membrane antigen"):ti,ab,kw	6,746
I-KQ1	**4**	#2 AND #3	3,807
P&I KQ1	**5**	#1 AND #4	3,503
O	**6**	response:ti,ab,kw	2,720,005
KQ1 limit	**7**	#5 AND #6	517
Final KQ1	**8**	#7 AND ('article'/it OR 'article in press'/it OR 'review'/it)	**203**

Cochrane Library; Date: 27 August 2020

PICOS	#	Search queries	Articles #
P	**1**	(prostat* AND (tumor OR tumour OR cancer OR carcinoma OR malignan* OR neoplasm*))	15,920
	**2**	MeSH descriptor: [Prostatic Neoplasms] explode all trees	5,472
P-KQ1	**3**	#1 OR #2	15,920
I	**4**	(“positron emission tomography” OR PET OR “PET CT” OR PET*CT OR PET-CT OR “PET MRI” OR “PET MR” OR PET*MRI OR PET*MR OR PET-MRI OR PET-MR)	8,050
	**5**	MeSH descriptor: [Positron-Emission Tomography] explode all trees	980
	**6**	#4 OR #5	8,050
	**7**	(PSMA OR "prostate specific membrane antigen")	226
I-KQ1	**8**	#6 AND #7	146
P&I-KQ1	**9**	#3 AND #8	144
O-KQ1	**10**	response	232,215

KQ1 limit	**11**	#9 AND #10	48
Final KQ1	**12**	#16 AND Cochrane Review, Trial	45
